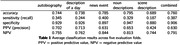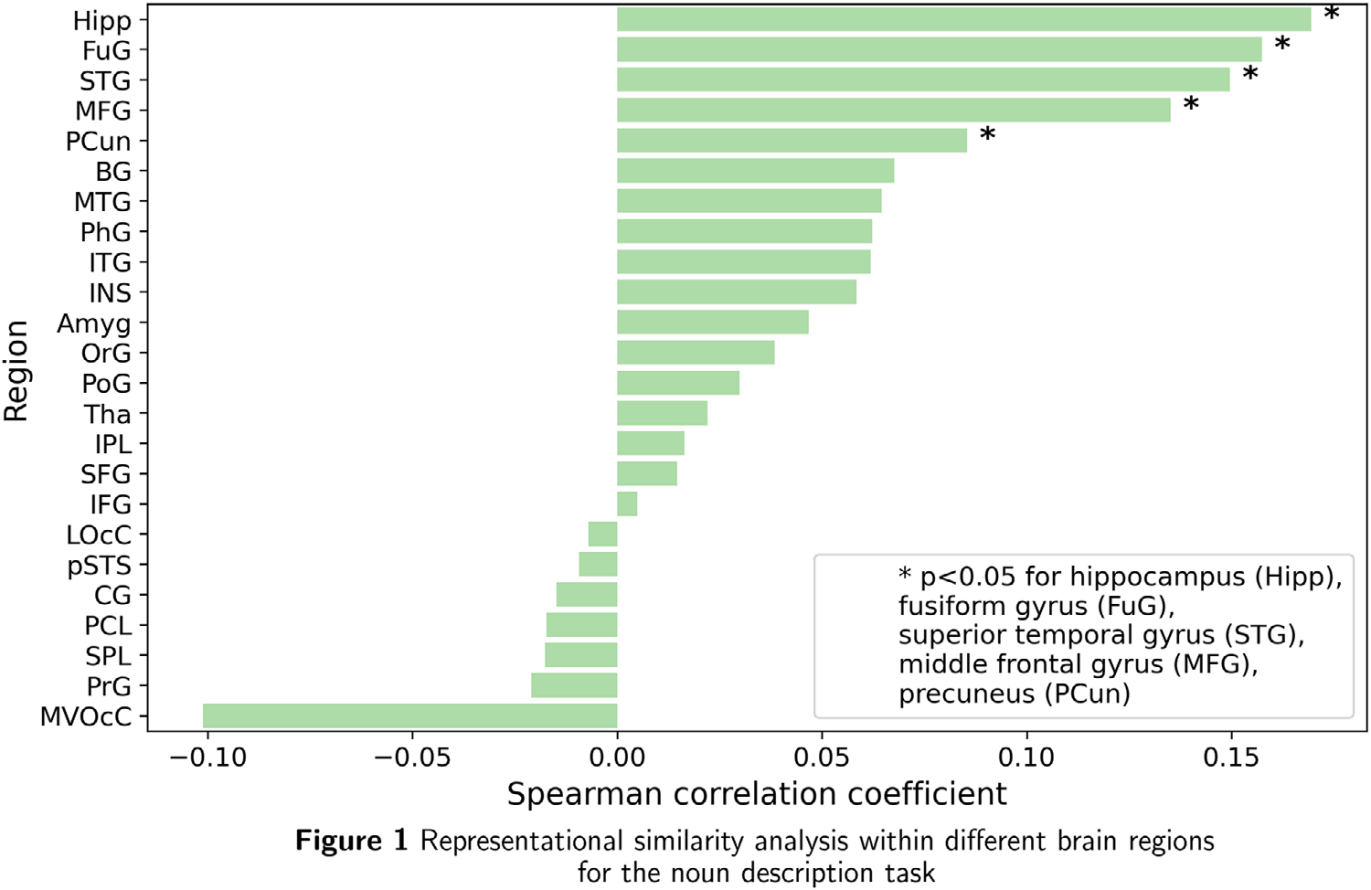# Natural language processing‐based analysis of connected speech in prodromal Alzheimer’s disease

**DOI:** 10.1002/alz.091238

**Published:** 2025-01-03

**Authors:** Helena Balabin, Laure Spruyt, Ella Eycken, Ines Kabouche, Bastiaan Tamm, Jolien Schaeverbeke, Patrick Dupont, Marie‐Francine Moens, Rik Vandenberghe

**Affiliations:** ^1^ KU Leuven, Leuven Belgium

## Abstract

**Background:**

Connected speech has been explored as a possible marker for Alzheimer’s disease (AD) by employing language models based on machine learning. However, most previous approaches are based on scene description tasks, and it is unclear how different types of connected speech and differences across subjects’ speech relate to changes in their brains.

**Method:**

We analyzed transcripts of Flemish Dutch connected speech from interviews from 74 cognitively healthy elderly adults (mean MMSE = 28.71 [25‐30], age = 73.15 years, 40 female) and 27 prodromal AD patients (mean MMSE = 25.22 [20‐30], age = 71.64 years, 13 female) across five parts: an autobiography, descriptions of a recent day, a news event, a set of nouns and the Cookie Theft scene. In the noun description task, subjects were asked to explain 10 abstract and 10 concrete nouns in detail. Using the Dutch RobBERT language model, we derived speech‐based representations for each interview part and subject, and first differentiated the two groups by fine‐tuning RobBERT with a classification layer. Then, we linked speech‐based features from the best performing task and structural MRIs for 99 subjects. Normalized gray matter volumes were processed using the Brainnetome atlas, resulting in 246‐dimensional subject representations, separated by regions. Distance matrices across subjects for speech‐ and MRI‐based representations were correlated in a representational similarity analysis (RSA), yielding Spearman correlations for each brain region.

**Result:**

For the classification task (see Table 1), all interview parts exceeded the majority class baseline accuracy of 0.733, except for the scene descriptions. The noun descriptions resulted in the highest accuracy (0.795) and specificity (0.947) across the interview parts. Overall, classification yielded high specificity but low sensitivity. Further, the RSA correlation between the subjects’ noun descriptions and their gray matter volumes can be mainly attributed to the hippocampus, fusiform gyrus (FuG) and superior temporal gyrus (STG) (see Figure 1).

**Conclusion:**

Connected speech elicited by scene description tasks reveal low accuracy and sensitivity for automated natural language processing‐based detection of prodromal AD. Noun descriptions could emerge as a viable alternative, demonstrated by their improved classification performance and link to brain regions relevant for memory and language.